# Nitrogen deficiency impacts on leaf cell and tissue structure with consequences for senescence associated processes in *Brassica napus*

**DOI:** 10.1186/s40529-016-0125-y

**Published:** 2016-05-24

**Authors:** Clément Sorin, Laurent Leport, Mireille Cambert, Alain Bouchereau, François Mariette, Maja Musse

**Affiliations:** 1 Irstea, UR OPAALE, 35044 Rennes, France; 2 INRA, UMR 1349, Institut de Génétique, Environment et Protection des Plantes (IGEPP), UMR INRA-Agrocampus Ouest-Université de Rennes 1, 35653 Le Rheu Cedex, France; 3 Univ Bretagne Loire, Rennes, France

**Keywords:** Oilseed rape, Leaf senescence, NMR Relaxometry, Transverse relaxation (T_2_), Microscopy

## Abstract

Improvement of nutrient use efficiency is a major goal for several crop plants, especially *Brassica napus*. Indeed, the low nitrogen use efficiency (NUE) in this crop results in negative economic and ecological consequences. The low NUE of oilseed rape is mainly due to low remobilization of nitrogen from vegetative parts to growing organs. Remobilization of leaf nitrogen takes place during senescence, a process known to strongly modify cell and tissue structure. This study focused on the impact of moderate N depletion, expected to induce 30 % reduction of seed yield, on these structural modifications. Two genotypes (Aviso and Express) were studied, with different tolerance of nitrogen depletion, evaluated through seed yield and dry mass production. Structural modifications of leaf cells and tissues were investigated through NMR relaxometry and light microscopy. Lower tolerance of N depletion was associated with higher impact on senescence associated structural modification pattern. The link between leaf structure modifications and nutrient remobilization is discussed. It is proposed that leaf structure monitoring during senescence through NMR device could be developed to select genotypes with high NUE.

**Electronic supplementary material:**

The online version of this article (doi:10.1186/s40529-016-0125-y) contains supplementary material, which is available to authorized users.

## Background

*Brassica napus* is a worldwide crop with numerous uses in food, feed and non-food products (biofuel, lubricants, etc.). Its production has increased more than five-fold over the last 30 years (www.fao.org). Nitrogen (N) fertilization is one of the highest costs of oilseed rape production (Singh 2005). Conventional crop management practices requires the use of relatively high amounts of N fertilizers (from 150 to 300 kg of N ha^−1^) to ensure an optimum yield (Rathke et al. 2006). Whatever the rate of N fertilization, the oilseed rape N harvest index is low compared to cereals (Dreccer et al. 2000) and, despite a good capacity for N absorption, less than 50 % of the nitrogen absorbed by the plant is present in the seeds at harvest (Schjoerring et al. 1995). Oilseed rape is known to have low nitrogen use efficiency (NUE), mainly due to low N remobilization efficiency (NRE) during sequential leaf senescence, as reported in several studies performed under field and controlled conditions (Malagoli et al. 2005; Gombert et al. 2006; Tilsner et al. 2005). As reviewed by (Avice and Etienne 2014), the remobilization processes associated with senescence in this crop species are not maximal and can lead to high amounts of residual organic nitrogen in falling leaves. In addition, this low remobilization efficiency during sequential senescence strongly impacts on agronomic potential and final yield (Noquet et al. 2004). Its low NRE therefore affects oilseed rape production both economically and environmentally (Singh 2005). Some studies have focused on improving other components of the NUE, e.g., nitrogen uptake efficiency (NupE) (Schulte auf’m Erley et al. 2007) and nitrogen assimilation efficiency (NAE) (Good and Beatty 2011). However, greater NRE has been considered a major target in the current context of Nitrogen input reduction, in order to maintain oilseed rape yield (Berry et al. 2010; Kessel and Becker 1999; Miro 2010).

Senescence processes have been shown to be highly regulated (Buchanan-Wollaston et al. 2003) and former studies have demonstrated that numerous differentially regulated genes are involved in nitrogen metabolism and remobilization processes (Horst et al. 2003; Guiboileau et al. 2012). A high NRE should fit with an optimum rate of N recycling, originating from the dismantling of plastids, other organits and macromolecules (Martínez et al. 2008; Avila-Ospina et al. 2014). Plastids represent around 15 % of the volume of oilseed rape leaves and are the main source of N and C. Vacuoles, representing the main site of macromolecule degradation during senescence, are also highly affected by remobilization in terms of enzymatic activity (Otegui et al. 2005). Moreover, the vacuolar volume of oilseed rape leaves have also been shown to be modified during senescence, with significant changes in the tissue structure (Sorin et al. 2015). Indeed, the cell wall seems to be modified during natural senescence while leaf water content increases, leading to an increase in cell size, especially in the palisade parenchyma. The low NRE of oilseed rape during the vegetative stages might not be due to limited amino acid transport to the phloem but seems to be related to incomplete hydrolysis of proteins (Noiraud et al. 2003; Tilsner et al. 2005). The regulation and efficiency of the degrading enzyme and autophagic process during senescence has been extensively studied (Martinez et al. 2008; Sakamoto 2006), highlighting the upregulation of numerous proteases (Gombert et al. 2006).

Cellular and tissular modifications during sequential leaf senescence can be evaluated finely by low field proton nuclear magnetic resonance (NMR), that has been used for investigation of cell water compartmentalization in various plant organs (Hills and Remigereau 1997; van der Weerd et al. 2001; Musse et al. 2010; Duval et al. 2005). The NMR technique allows measurement of relaxation signals that for hydrated plant tissues originate mainly from water. For plant tissues, characterized by compartmentalized cells with relatively slow diffusion exchange of water molecules between compartments, the relaxation times are multi-exponential due to differences in physical and chemical properties of water in different compartments. The transverse relaxation time (T_2_), particularly sensitive to variations in water properties occurring in plant tissues, is therefore used to study changes in water status and distribution. NMR has been recently used to investigate senescence process in oilseed rape leaves (Musse et al. 2013; Sorin et al. 2015). An interpretation of the multi-exponential NMR signal of leaf tissue taking into account both cellular compartmentalization and heterogeneities at tissue level has been proposed on the bases of the NMR experiments, micrographs and physiological leaf characteristics. According to these studies, the fastest relaxing component (relaxing at around a few dozen µs) has been associated with the protons from dry matter. Among the water-associated components, the shortest T_2_-component (about few ms) has been assigned to apoplastic water and to a lesser extent to water inside starch granules, the component relaxing with T_2_ of about a few dozen ms to the plastidial water and/or different proton pools such as water in senescence-associated vacuoles etc. The longest T_2_-component in young leaves has been related to vacuole water and was demonstrated to split into two components in old leaves as a result of hydration and cell enlargement associated to senescence processes.

The previous results highlighted the importance of considering the complexity of the tissue structure while studying leaf functioning. In order to go a step further in the investigation of the senescence process, we investigated the effects of nitrogen deficiency on modifications of tissue and cell structure occurring in oilseed rape during senescence. Indeed, this abiotic constraint is well known to induce major modifications in leaf senescence process (Avice and Etienne 2014) and to interfere with nutrient remobilization (Albert et al. 2012), with a strong effect on seed yield (AlJaloud et al. 1996; Andersen et al. 1996). This study will therefore aim to investigate by NMR the impact of N deficiency on the senescence process and its link with yield reduction. An additional output of this study would be to evaluate NMR as a new method for N nutrition investigations.

Two genotypes of oilseed rape contrasting in terms of the response to nitrogen stress were studied: the Aviso genotype was chosen because it is known to be adapted to nitrogen depletion (Bouchet et al. 2014) while the Express genotype is known to be more affected by N stress (Rathke et al. 2006). Plants were grown in a controlled environment reproducing optimal field conditions and were submitted to moderate N deficiency expected to induce 30 % reduction of seed yield. Leaves of plants of both genotypes were sampled during stem elongation, a period in which remobilization from senescing organs is significant. Indeed, several studies have shown that leaves at this stage can be considered the major contributors of N and C to the seeds (Noquet et al. 2004; Malagoli et al. 2005). Leaf structure modifications were revealed by low field NMR and light microscopy. The physiological status of leaves was characterized through chlorophyll fluorescence, chlorophyll, dry matter and water content. The N depletion treatment was also assessed at final harvest time by evaluation of seed yield (SY) and shoot dry mass (DM) production.

## Methods

### Plant material

Twenty oilseed rape seeds of each Aviso and Express genotype were sown in containers filled with a growing medium (FALIENOR 9226-6F2) containing 65 % light peat, 20 % dark peat and 15 % perlite. Eight homogenous three-day-old seedlings of each genotype were individually planted into 4-l pots filled with the same medium and grown in a growth chamber. Growth chamber conditions were 14 h daylight (at 200 µmol photons m^−2^ s^−1^) and 10 h dark (relative humidity: 80 %; temperature: 22 °C/17 °C). After 6 weeks in the growth chamber, the newest leaf of each plant was tagged and referred to as rank 0. At that time, the temperature was decreased over 1 week to +4 °C and the plants were then submitted to an 8-week vernalization period (+4 °C). At the end of this period, the conditions were returned to the initial values over 1 week. Before vernalization all plants were supplied with 177.5 mg of N for each plant and control plants received an additional 70 mg of N at the end of the vernalization period. The N-deficient plants did not receive any supplementary N. Leaves were sampled 4 weeks after the end of the vernalization period. Taking into account the potential mineralization of the soil during the growth period, the aim of this N-treatment was to provide per plant the quantity of N available in crop conditions for control plants, with a reduction for N-depleted plants that induce a minimum of 30 % seed yield reduction.

All measurements were performed on the five to six oldest leaves of each plant. To do so, one to two older and three younger leaves than the reference senescing leaf (rank 0) from Aviso and Express plants grown under optimal and sub-optimal nitrogen fertilization were analyzed.

### Chlorophyll content and fluorescence yield

One day before sampling, relative chlorophyll content per unit of leaf area was estimated using a non-destructive chlorophyll meter SPAD (Soil Plant Analysis Development; Minolta, model SPAD-502). The chlorophyll content of each leaf was estimated as an average value of 6 independent measurements and was expressed as a percentage of the highest value measured across the leaf rank of the plant. In addition to chlorophyll content, chlorophyll fluorescence yields (Fv/Fm) were measured on all leaves studied using a portable chlorophyll fluorometer (Hansatech Handy PEA). Measurements were carried out near the central vein, after a dark adaptation time of 10 min.

### NMR relaxometry

Depending of the leaf size, six to eight discs of 8 mm in diameter were cut from fresh limb tissue for the NMR experiment. In order to obtain homogeneous tissues, discs were taken from each side of the central vein, as close as possible to the vein and avoiding lateral second order veins. Discs were then placed in NMR tubes which were closed with a 2-cm long Teflon cap to avoid water loss during measurements.

NMR Relaxometry measurements were performed with a 20 MHz spectrometer (Minispec PC-120, Bruker, Karlsruhe, Germany) equipped with a thermostatted probe. Temperature was set at 18 °C. Transverse relaxation time (T_2_) was measured using the combined FID-CPMG sequence.

The FID signal was acquired from 11 µs to 70 µs at a sampling decay of 0.4 µs. For the CPMG measurements, the 90°–180° pulse spacing was 0.1 ms and the signal of a single point was acquired at the echo maximum. Data were averaged over 64 acquisitions. The number of successive echoes recorded was adjusted for each sample according to its T_2_. The recycle delay for each sample was adjusted after measurement of the T_1_ with a fast-saturation-recovery sequence. The total time of acquisition of data for T_2_ (including spectrometer adjustments and T_1_ measurement) was about 10 min per sample.

Fitting of the CPMG signal was performed using Scilab software according to the MEM (Mariette et al. 1996), which provides a continuous distribution of relaxation time components without any assumption concerning their number. In this representation, the peaks of the distribution are centered at the corresponding most probable T_2_ values, while peak areas correspond to the intensity of the T_2_ components. The T_2_ and intensity of the FID signal (corresponding to the protons of the solid part) were computed from the signal of the combined FID-CPMG sequence using the Lavenberg-Marquardt algorithm which allows a discrete solution for the fitting curve according to the equation:

1 $$I(t) = I_{1} \exp ( - t/T_{21} )^{2} \, {+} \sum\limits_{i = 2} {I_{0i} \exp (} - t/T_{21} ) + offset$$

where I_0i_ is the intensity of the ith exponential at the equilibrium state and T_2i_ the characteristic transverse relaxation time for the ith exponential. Signal intensity was expressed through the specific leaf water weight of the ith signal component (LWW) expressed in g m^−2^. The specific LWW of each CPMG component was calculated according to the equation:

2 $$LWW_{i} = \frac{{I_{R0i}\, m_{w} }}{A}$$

where m_w_ is the water mass of the leaf disc used for NMR (in g), A the leaf disc area (in m^2^) and I_R0i_ the relative intensity of the ith signal component expressed as a percentage of the total CPMG intensity.

### Water content

Water content (WC) was measured on all leaf discs sampled for NMR relaxometry by weighing before (fresh weight) and after drying (dry weight) in an oven at 70 °C for 48 h. WC was expressed as percentage of fresh weight.

### Light microscopy

For microscopy studies, mesophyll tissues from the leaves studied were collected from control and stressed plants of both genotypes. For each of the four conditions, one mature (leaf rank 0) and one senescent (lowest leaf rank available) were sampled and three to five bands 3-mm wide and 1-cm in length were cut perpendicularly to the central vein. This protocol has been applied on two biological replicates. The leaves were included as described by Sorin et al. (2015). From each band, thin Sects. (1 µm) were cut with an ultramicrotome (LEICA RM 2165) and stained with toluidine blue before they were observed with a NIKON Eclipse 80i microscope. The numerical results of each leaf analyzed resulted from about 400 cells (200 per parenchyma) observed from 4 to 10 images.

Image J software was used for the analyses of the micrographs. The area (A) and width (W) of each cell were measured from both palisade and spongy parenchyma and vacuolar volume was then computed by multiplying A by W. The thickness of the leaf and of the individual (palisade and spongy) tissues were also measured form the images. Finally, the intercellular spaces were manually estimated using the following method. It consists in finding the threshold values in HSB color space that segments the colored image into cell walls and background. Intercellular spaces are then filled with a single gray level with the Flood Fill Tool and then quantified. The volume fraction of intercellular spaces was expressed as percentage of the leaf surface analyzed.

### Data analysis

Statistical analyses were performed using the R software (R version 2.15.1). ANOVA was performed for each trait to test for growth conditions, genotype, leaf rank and the interaction effects. Comparison of the means using the multiple range LSD (Fisher’s Least Significant Difference) test was performed between the different leaf ranks of the same growth condition and between the growth conditions for the same leaf rank. Correlations were revealed through Pearson’s rank correlation coefficient analysis.

## Results

### Characterization of leaf ageing and senescence

**Fig. 1:**
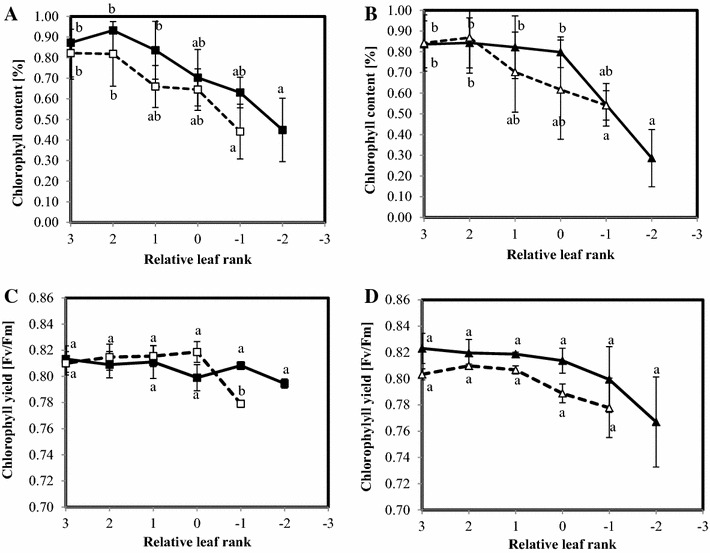
Chlorophyll content (**A**, **B**) and yield (**C**, **D**) of the oldest leaves of 18-weeks old plants of two oilseed rape genotypes, Aviso (**A**, **C**) and Express (**B**, **D**), grown in two conditions: control (*solid line*) and nitrogen deficiency (*dotted line*). Each data point corresponds to the average of four repetitions. Relative leaf rank 0 corresponds to the last leaf appearing before vernalization.* Different letters* mean significant (p < 0.05) differences between leaf rank of the same condition and stars highlight significant differences between conditions for the same leaf rank

**Fig. 2:**
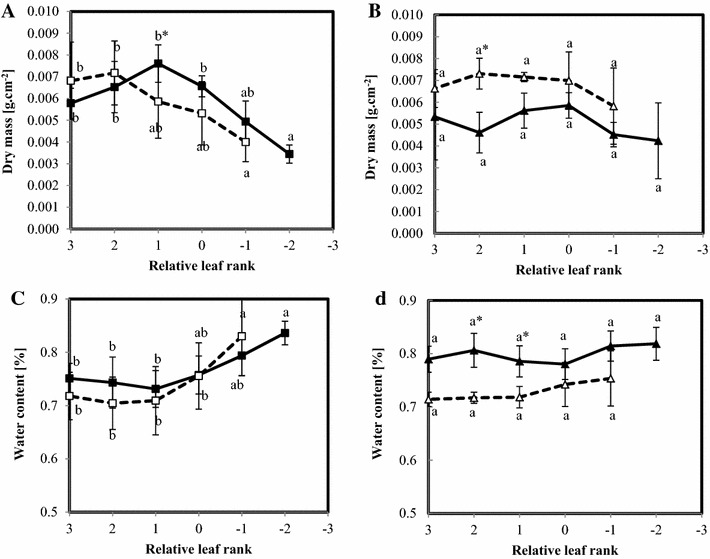
Dry mass per disc (**A**, **B**) and water content (**C**, **D**) of the oldest leaves of 18-weeks oilseed rape plants of two genotypes, Aviso (**A**, **C**) and Express (**B**, **D**), grown in two conditions: control (*solid line*) and nitrogen deficiency (*dotted line*). Each data point corresponds to the average of four repetitions. Relative leaf rank 0 corresponds to the last leaf appearing before vernalization.* Different letters* mean significant (p < 0.05) differences between leaf rank of the same condition and stars highlight significant differences between conditions for the same leaf rank

As described in the “Methods” section, the measurements were performed on the five or six oldest leaves. Rank 0 corresponded to the last leaf appeared on each plant before the beginning of the vernalization period, thus positive ranks corresponded to leaves appeared during vernalization. For both genotypes, nitrogen stress induced earlier leaf fall than in well fed plants, as can be seen in Figs. 1 and 2.

The impact of nitrogen deficiency on photosynthetic apparatus was evaluated through both chlorophyll content and chlorophyll fluorescence yield (Fig. 1). As expected, all plants presented a decrease in chlorophyll content with ageing. Nitrogen depletion induced a decrease in chlorophyll content (Fig. 1A, B) in all leaves of the Aviso plants, while this trend was less marked for express leaves. Chlorophyll fluorescence yield remained stable for all the Aviso leaves studied whatever the N treatment (Fig. 1C). In contrast, chlorophyll fluorescence yield decreased slightly with both age and nitrogen treatment in leaves from the Express genotype (Fig. 1D). Dry matter per leaf area decreased according to leaf age in Aviso, and remained stable in Express leaves (Fig. 2A, B). Nitrogen depletion had a different impact on dry matter per leaf area in the two genotypes investigated, with a reduction in Aviso and a rise in Express plants. Water content (Fig. 2C, D) in the youngest leaves (rank 3 to rank 1) in the Aviso plants grown in both optimal and low nitrogen conditions was relatively low (around 70 % of fresh weight), and it increased with leaf age to 85 % for the oldest leaves studied. Water content remained constant and clearly distinct between treatments in Express leaves (around 80 % for control and 70 % for stressed leaves).

### Structural modifications

**Fig. 3:**
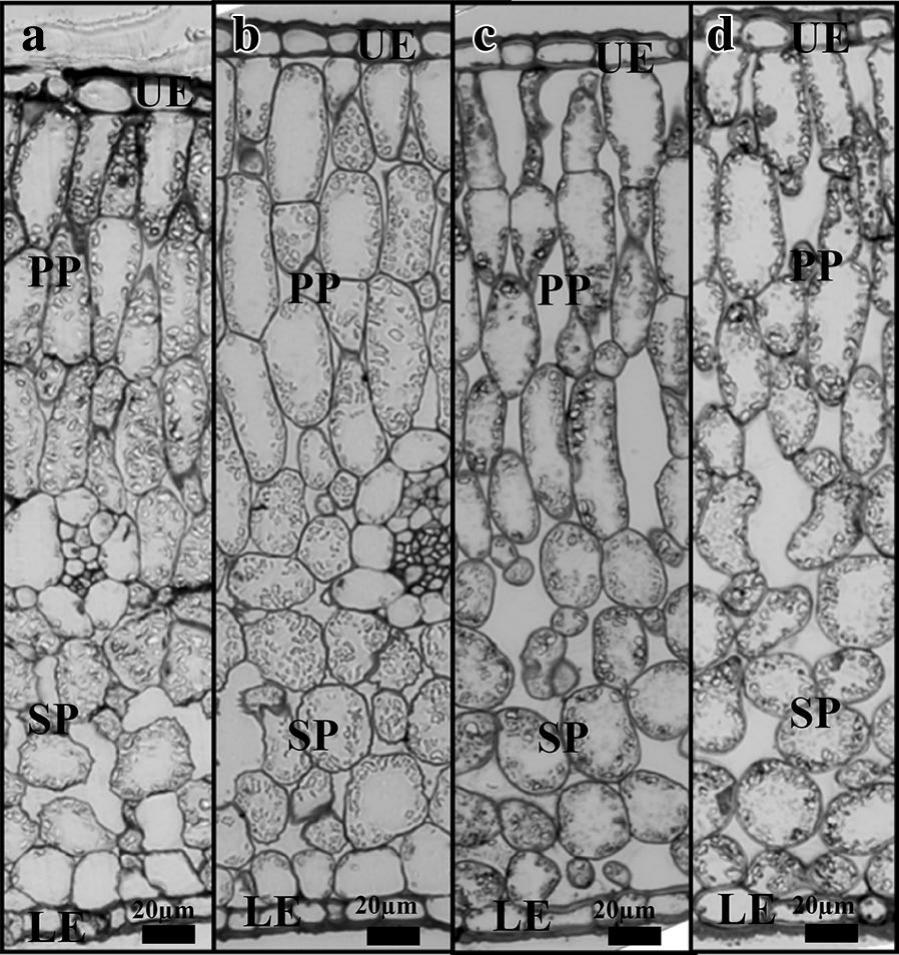
Micrographs of cross sections from the two oldest leaves of Aviso genotype in both conditions. **a** control condition rank 0; **b** control condition rank −2; **c** N deficiency condition rank 0; **d** N deficiency condition rank −1. *UE* upper epidermis, *PP* palisade parenchyma, *SP* spongy parenchyma, *LE* lower epidermis

**Fig. 4:**
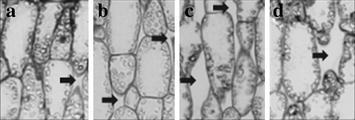
Enlargement of micrographs corresponding to the cross sections of leaves (Aviso genotype) showing palisade cells. **a** control conditions, rank 0; **b** control conditions, rank −2; **c** N deficiency conditions, rank 0; **d** N deficiency condition rank −1. *Arrows* show intercellular spaces

**Fig. 5:**
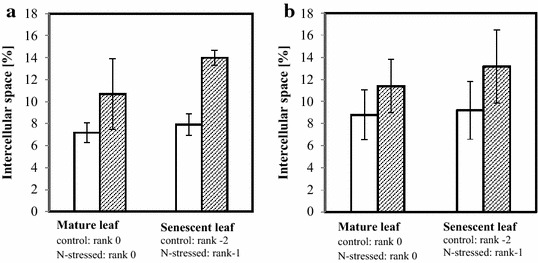
Percentage of intercellular space in mesophyll of Aviso (**a**) and Express (**b**) leaves from plants grown in control (*white*) and N deficiency conditions (*hatched*)

Figure 3 shows light micrographs of leaf tissues corresponding to different leaf ranks from control and N-stressed Aviso plants. Palisade parenchyma consisted of three to four layers of regular-shaped cells while spongy parenchyma was characterized by less organized structure. No significant differences in leaf thickness were observed on the leaf micrographs between the youngest and oldest leaves studied from plants grown in both control and stressed conditions. Epidermis tissues were very thin but covered with a relatively thick wax layer. As expected, intercellular spaces were mainly present in spongy parenchyma leaves from control plants (see Fig. 3), although some spaces were observed in palisade parenchyma, as already reported by Evans and von Caemmerer (1996). More surprisingly, significantly higher number of large intercellular spaces were observed in both palisade and spongy parenchyma in leaves from stressed plants compared to control. For palisade parenchyma, that change is illustrated by the Fig. 4 that also shows the increase in size of palisade cells with ageing. The Fig. 5 corresponds to the percentage of intercellular spaces measured in mature and senescent leaves for both conditions and for both genotypes. The effects of N depletion seemed to be greater with ageing. The same trends were observed for the Express plants, but to a lesser extent (Additional file 1: Figure S1, Fig. 5). Chloroplasts were visible in all leaves and did not significantly differ in number whatever the leaf age, indicating that in the conditions of the present study the senescence process was far from being complete even in the oldest leaves studied.

**Fig. 6:**
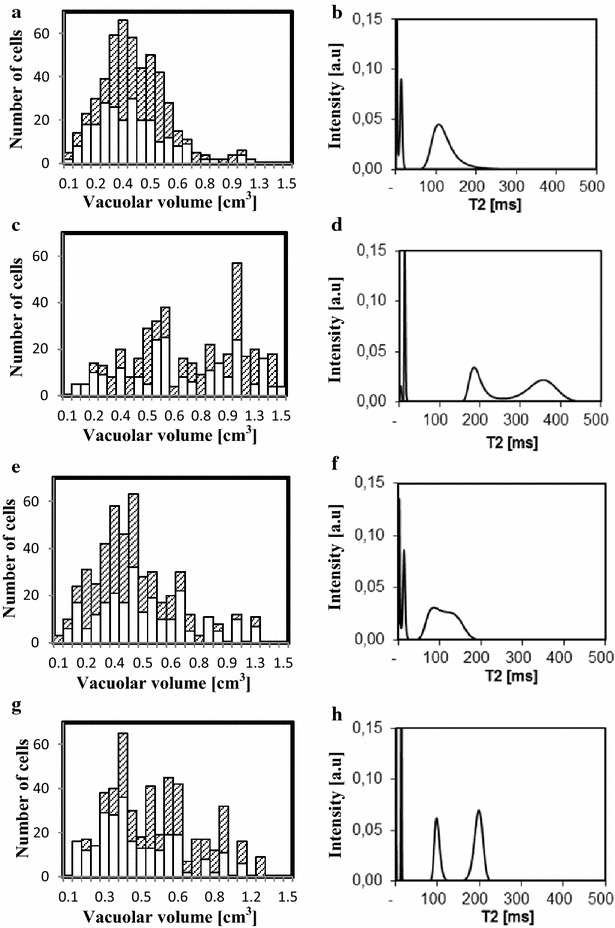
Vacuolar volume distribution (**a**, **c**, **e**, and **g**) and transverse relaxation time distribution (**b**, **d**, **f**, **h**) of two old leaves of an Aviso plant in control (**a**–**d**) and N deficiency conditions (**e**–**h**). **a** and **b** correspond to rank 0, **c** and **d** to rank −2, **e** and **f** to rank 0 (in stressed condition) and **g** and **h** to rank −1 (in stressed condition). In the vacuolar volume distributions *white bar* corresponds to spongy vacuoles and hatched bar to palisade vacuoles

The distribution of vacuolar volume in all cells of both the palisade and the spongy parenchyma of Aviso leaves is presented in Fig. 6 (a, c, e and g). For control plants, the results indicated that in the majority of cells the vacuolar volume of the rank 0 leaf (Fig. 6a) was centered at about 0.35 cm^3^. With ageing (leaves of rank −2, Fig. 6c), the distribution of vacuolar volume was wider, indicating an increase in the number of large cells. The same trend was observed in leaves from N-depleted plants (Fig. 6e, g). Figure 6 (b, d, f and h) depicts a continuous distribution of water NMR transverse relaxation times obtained from the same leaves as presented in Fig. 6a, c, e and g. Three components, relaxing at around 2, 12, and 100 ms, were observed for the mature leaves (rank 0) of plants grown in both conditions (Fig. 6b, f). The longest T_2_ component attributed to the vacuole (Musse et al. 2013; Van As 2007) represented more than 75 % of the leaf water. The T_2_ of this component split into two components in the oldest leaves analyzed (Fig. 6d, h), resulting in two separate vacuolar components, as already described (Sorin et al. 2015).

The relatively homogeneous distribution of vacuole volumes found in the youngest leaves studied (Fig. 6A, E) may be associated with a single vacuole-associated NMR signal component (Fig. 6E, F). On the other hand, enlargement of some cells from both parenchyma observed in senescing leaves (Fig. 6C, G) was in agreement with the splitting of the longest T_2_ NMR signal (Fig. 6 D and F).

It is of note that, in addition to the water NMR signal components, a fast relaxing component was measured associated with the protons from dry matter, relaxing at around 0.03 ms. This represented about 5 % of the total signal intensity, and the intensity of this component decreased with ageing as the dry mass of the leaf decreased.

**Fig. 7:**
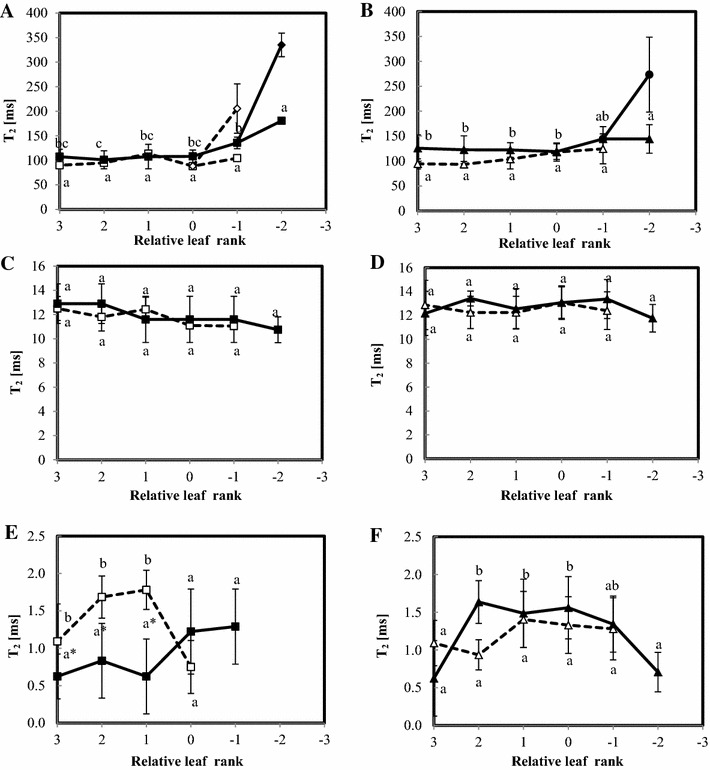
T_2_ of leaves from Aviso (**A**, **C**, **E**) and Express (**B**, **D**, **F**) genotypes in control (*solid line*) and N deficiency (*dotted line*) conditions. Fourth and fifth components of the NMR signal are presented in **A**, **B**, the third in **C**, **D** and the second in **E**, **F**.* Different letters* mean significant (p < 0.05) differences between leaf rank of the same condition and *stars* highlight significant differences between conditions for the same leaf rank

Figure 7 shows the T_2_ values of the water-associated NMR signal (components 2 to 5) during leaf ageing for control and stressed plants of both Aviso and Express genotypes. In both cases, the fourth NMR signal component was about 100 ms in higher leaf ranks (rank 3 to rank 0). In control plants, the signal then started to increase and split for the oldest leaves studied (rank −2) as shown in Fig. 6. Nitrogen depletion accelerated the appearance of the fifth NMR signal component for Aviso leaves, whereas no differences were observed between the two N conditions in Express leaves (Fig. 7A, B). The T_2_ of the third component remained stable for both genotypes and both conditions (Fig. 7C, D). The second component disappeared in the oldest leaves (rank −2) of control Aviso plants, while it was observed for all leaves of control express plants (Fig. 7F). Moreover, the pattern of evolution of the T_2_ of this second component differed between conditions. In Aviso stressed plants, mature leaves presented higher T_2_ than control leaves (ranks 3 to 1), whereas there were no significant differences in Express plants.

**Fig. 8:**
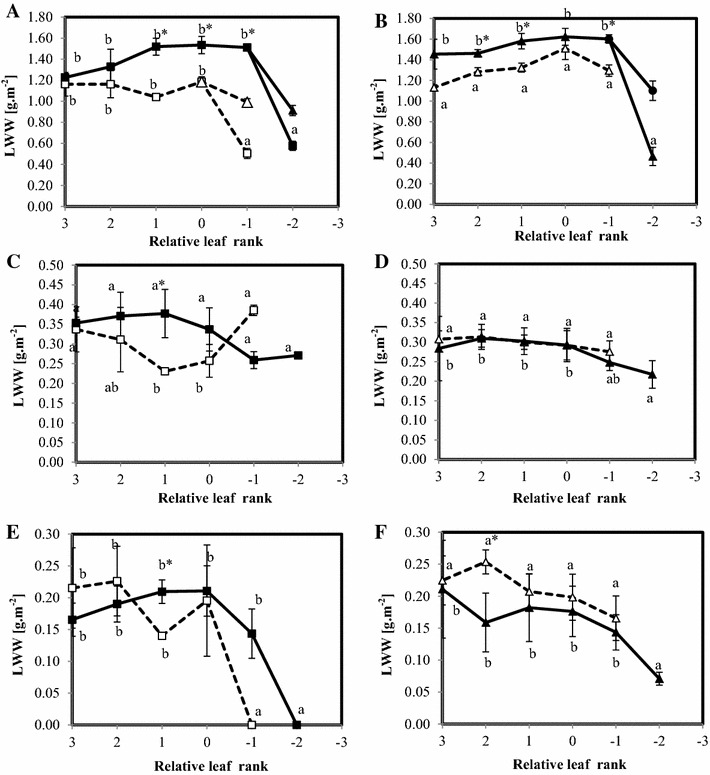
LWW (expressed in g m^−2^) of leaves from Aviso (**A**, **C**, **E**) and Express (**B**, **D**, **F**) genotypes in control (*solid line*) and N deficiency (*dotted line*) conditions. Fourth and fifth components of the NMR signal are presented in **A**, **B**, the third one in **C**, **D** and the second component **E**, **F**.* Different letters* mean significant (p < 0.05) differences between leaf rank of the same condition and *stars* highlight significant differences between conditions for the same leaf rank

Figure 8 represents the amount of water corresponding to the different components of the NMR signal of leaves (LWW) according to nodal position in Aviso and Express plants. For both genotypes, the LWW of the fourth component of control plants increased very slightly, ranging from about 1.2 g/m^2^ in young leaves to 1.5 g/m^2^ in senescing leaves. In the oldest leaves (rank −2), the LWW of the fourth and fifth components (LWW_4+5_), generated by the split, was around 1.5 g/m^2^, and about two thirds of this water was associated with component 5. The LWW value of the third component was almost constant for all leaf ranks and genotypes and for both N treatments. The LWW values of the second component were stable at around 0.2 g/m^2^ in the youngest leaves of Aviso plants and decreased when the split occurred, reaching zero in the oldest leaves. The same evolution was observed in Express plants but to a lesser extent. The N treatment did not affect the values in Aviso plants whereas a slight increase in LWW_2_ was observed for all leaves of the Express plants.

## Discussion

### Impact of moderate nitrogen deficiency on phenological and physiological traits

The reduction in N fertilization was conducted in the present study in such a way as to correspond to the reduction in N input under field conditions envisaged in the context of near future sustainable oilseed rape production (Singh 2005). As shown through the several parameters measured, this relatively moderate nitrogen fertilization impacted plants in terms of phenological and physiological traits. Indeed, for both genotypes, in accordance with previous findings (Gironde et al. 2015), a reduction in number of leaf ranks was observed in stressed plants, corresponding to a disturbance of plant development (Additional file 2: Figure S2) (Albert et al. 2012; Gombert et al. 2006; Masclaux-Daubresse et al. 2008). In the case of the Aviso genotype, nitrogen depletion may also have affected the growth of young leaves, as already reported in the literature (Bouchet et al. 2014). The other physiological parameters measured, i.e, water content, dry matter and indicators of photosynthesis performance (chlorophyll fluorescence yield and content) were not significantly impacted by N depletion, indicating that the study was focused on an early phase of nitrogen deficiency. Indeed, these parameters are known to be strongly affected by long-term N stress (Gombert et al. 2006). Moreover, the measurements were performed at the onset of post-vernalization regrowth when, on the basis of their physiological status, the oldest leaves seemed to fall before completing the senescence process. The results of physiological measurements showed an expected rank to rank senescence pattern (decrease in chlorophyll content and dry mass, Figs. 1 and 2) in both genotypes, clearly indicating that the sampling procedure followed the senescence associated remobilization process from its beginning to the leaf falling. In addition to the physiological impact, the effects of N stress were also observed on phenological and final yield characteristics as described below. Indeed, N stress induced a delay in flowering time and a reduction in biomass production and seed yield measured on plants until maturity and harvest time.

### Structural modifications caused by N deficiency

The palisade parenchyma in oilseed rape leaves consists of regular shaped cells organized in layers, whereas the spongy parenchyma is less well organized and presents large intercellular spaces (Castro-Diez et al. 2000). These two predominant tissues are surrounded by epidermis cells and covered by cuticles. Two palisade cell layers were reported in a previous study performed on oilseed rape of the Tenor genotype grown under non-vernalized conditions (Sorin et al. 2015). These results differed from those of the present study in which three to four layers were observed. Such structural differences are probably due to the effects of the increase in cell division stimulated by vernalization (Manupeerapan et al. 1992) but may be also due to genotypic differences. It would be interesting to pursue studies on the effects of the cold stress on the leaf structure setting. The lack of increase in leaf thickness associated with leaf ageing observed here contrasted with previous studies (Sorin et al. 2015). This could be attributed to the fact that, under the present conditions, the senescence process was incomplete (plastids still present in older leaves, low water content…). Moreover, this phenomenon could also be associated with the hardening phenomenon resulting from the vernalization process; a relatively thick cuticle observed on the micrographs probably limited the enlargement of the palisade layer. Note that although the leaf thickness did not increase, the internal leaf structure evolved during the senescence process, with an increase in cell size and a decrease in number of parenchyma cells. One other interesting point was that the epidermis cells were small and their size did not vary with leaf ageing, in contrast to earlier results (Sorin et al. 2015). These results emphasize the strong impact of growth conditions on leaf structure and confirm the importance of taking these conditions into account when studying senescence.

The fact that the amount of leaf water (LWW in g/m^2^) in Aviso plants was significantly lower in N-stressed than in control plants, although the water content was the same, could be explained by a reduction in dry weight due to remobilization of cell material. Indeed, an increase in the relative percentage of intercellular spaces in leaves from 8 ± 3 to 14 ± 1 % induced by N starvation was estimated from micrographs. In addition, whereas intercellular spaces in control plants were observed mainly in spongy parenchyma, these spaces were present in the whole mesophyll of leaves from stressed plants. The same phenomenon was observed for Express leaves but to a lesser extent. The increase in the amount of intercellular space might be attributed to the loss of cell-cell adhesion due to a decrease in cell wall binding, as observed in fleshy fruits (Redgwell et al. 1997).

According to (Martínez and Guiamet 2014), leaf apoplast has a major role in both cell structure (cell wall) and nutrient remobilization due to several apoplastic enzyme activities. Indeed, N depletion acting on the synthesis of proteins (both in translation and transcription) could be at the origin of lower enzyme activity of apoplastic proteins. Moreover, various studies have reported that, despite the absence of differences in terms of cellulose and hemicellulose, nitrogen depletion clearly decreased the amount of lignin (Murozuka et al. 2014; Wilson and Lt 1978), and different cell wall expansin genes are upregulated in response to such stress (Scheible et al. 2004).

Previous studies have demonstrated the sensitivity of the NMR signal to both cell and tissue modifications in oilseed rape leaves during senescence, and one interpretation has been proposed taking into account both cellular compartmentalization and heterogeneity at the tissue level. Sorin et al. (2015) demonstrated that the splitting of the last NMR signal component measured in mature leaves (component 4) into two components was due to massive enlargement of palisade cells during senescence linked to increased water content. The results of the present study indicated that splitting of the NMR component was associated with an increase in vacuole volume of a relatively large number of cells distributed not only in the palisade parenchyma but also in the spongy parenchyma (Fig. 5). The present study confirmed the phenomenon of senescence-associated structural modifications previously reported (Musse et al. 2013; Sorin et al. 2015). Here the cell enlargement was not restricted to the palisade layer; we have also been able to observe this phenomenon in the spongy tissue. This difference was probably due to the vernalization process. Nevertheless, the development-induced changes in cell structure were clearly revealed by the NMR signal, confirming that the relaxation signal can be used as an indicator of senescence progression whatever environmental conditions.

The absence of variation in the third NMR signal component with leaf ageing that is generally attributed to plastids (Musse et al. 2013) and observed for both genotypes and conditions was in accordance with the preservation of plastid integrity estimated by micrographs and confirmed by physiological parameters. This contrasted with findings reported in the literature (Ghosh et al. 2001; Sorin et al. 2015), where most of the plastids disappeared at the end of senescence. The difference was probably due to the fact that leaves fell before complete senescence in the specific conditions of the present study.

The relationship between the decrease in dry mass due to ageing and remobilization processes and changes in the signal intensity of the first component confirms previous findings, indicating that the dry matter signal can be used as a reliable indicator of relative leaf age (Musse et al. 2013). Moreover the development of portable, non-destructive, NMR measurement could provide a way to estimate dry mass directly in the field without cutting the leaf.

### Genotype differences in terms of response to N deficiency

The two genotypes used in the present study were chosen for their different response to N deficiency. At the experiment time, only few developmental differences were observed between conditions but with time the developmental delay induced by N-starvation became clearer. For Aviso N-stressed plants the flowering was 4 days late and 11 days late for Express. In addition, the final yield components (dry matter production (DM) and seed yield (SY)) showed that they were similar in control conditions (DM 15–16 g/plant, SY 3–3.5 g/plant), while noticeable differences were observed for plants submitted to N depletion. Indeed, DM was by about 30 ± 3 and 35 ± 2 % lower for Aviso and Express plants, respectively. The differences between genotypes were even more pronounced for SY decrease of about 37 ± 3 % for Aviso and 48 ± 3 % for Express plants. The yield data confirmed the greater ability of Aviso plants to adapt to N starvation.

The harvest index (ratio between seed yield and plant dry matter) showed more effective nutrient use by Aviso in the situation of nitrogen deficiency. Indeed, while the harvest index was nearly the same for both genotypes under control conditions, it decreased from 21 ± 2 to 19 ± 3 % for Aviso and to 17 ± 2 % for Express plants. These results are in agreement with those obtained in a study comparing ten oilseed rape genotypes where it was reported that maintaining of leaf biomass production under low nitrogen conditions was related to a greater tolerance to N deficiency for Aviso than for Express plants (Gironde et al. 2015).

The differences in the response to N deficiency between the genotypes studied in terms of Nitrogen use efficiency were consistent with the evolution of leaf tissue structure observed during the experimental period (the re-growth period just after the vernalization stage). First, the micrographs showed a greater increase with ageing in the relative volume fraction of intercellular spaces induced by N deficiency in Express plants compared to Aviso plants (Fig. 5). This was probably related to the lower water content of stressed Express leaves. On the other hand, water content in Aviso leaves did not seem to be affected by N deficiency and increased with leaf ageing, as previously reported (Musse et al. 2013). Secondly, the vacuolar component of stressed Aviso leaves split in the oldest leaves analyzed, whereas this was not the case for Express leaves. As the splitting of the fourth NMR signal component has been associated to an early event in leaf senescence (Sorin et al. 2015), its absence indicates that either the senescence process was interrupted by leaf fall or it proceeded differently. This hypothesis was supported by the fact that the pattern of the second NMR signal component was different from expected pattern; indeed it was expected to disappear at the end of natural senescence (Musse et al. 2013) but was still observed in the leaves just before falling.

The results of the present study confirmed that leaf tissues structural changes occur during senescence, in agreement with the results of the previous studies (Musse et al. 2013). We additionally showed here that depending on the growing conditions, these structural changes are not necessarily tissue dependent. Indeed, in senescing leaves of vernalized plants, large cells were distributed not only in palisade but also in spongy parenchyma.

The present study demonstrated that the pattern of evolution of leaf structure during senescence in oilseed rape was affected by N depletion, especially in the case of the Express genotype less adapted to this stress. The impact of the N stress on the leaf structure pattern evolution was observed before the changes in physiological parameters could even be measured, supporting the previous finding that leaf structural changes are an early event of the senescence process. The differences in yield between the two genotypes indicated a link between modifications of leaf tissue structure and the remobilization processes. Further studies will focus on mechanisms linked to these leaf structural changes that promote N and C remobilization and their effects on seed yield. A step further will also be to evaluate the NMR relaxometry for assessment of the effects of N depletion on senescence for genotypes screening for better response to such abiotic stress.

## Additional files

**Additional file 1: Figure S1.** Micrographs of cross sections from two old leaves of Express genotype in both conditions. a, e: control condition rank 0; b, f: Control condition rank -2; c, g: N deficiency condition rank 0; d, h: N deficiency condition rank -1. a, b, c, and d represent the palisade parenchyma while e, f, g, and h the spongy parenchyma. UE: upper epidermis and LE: lower epidermis.

**Additional file 2: Figure S2.** Developmental stage of Aviso (black) and Express (grey) plants in control (solid line) and N-deficiency (dotted line) conditions. Experiments were conduct on 1/31.
