# Supplementary material for: Nitrogen deficiency impacts on leaf cell and tissue structure with consequences for senescence associated processes in Brassica napus
Source: Bot Stud. 2016 May 24;57:11. doi: 10.1186/s40529-016-0125-y (PMC5430559; doi:10.1186/s40529-016-0125-y)
Supplement: Supplementary file 1 — Additional file 1: Figure S1. Micrographs of cross sections from two old leaves of Express genotype in both conditions. a, e: control condition rank 0; b, f: Control condition rank -2; c, g: N deficiency condition rank 0; d, h: N deficiency condition rank -1. a, b, c, and d represent the palisade parenchyma while e, f, g, and h the spongy parenchyma. UE: upper epidermis and LE: lower epidermis. [file 40529_2016_125_MOESM1_ESM.docx]

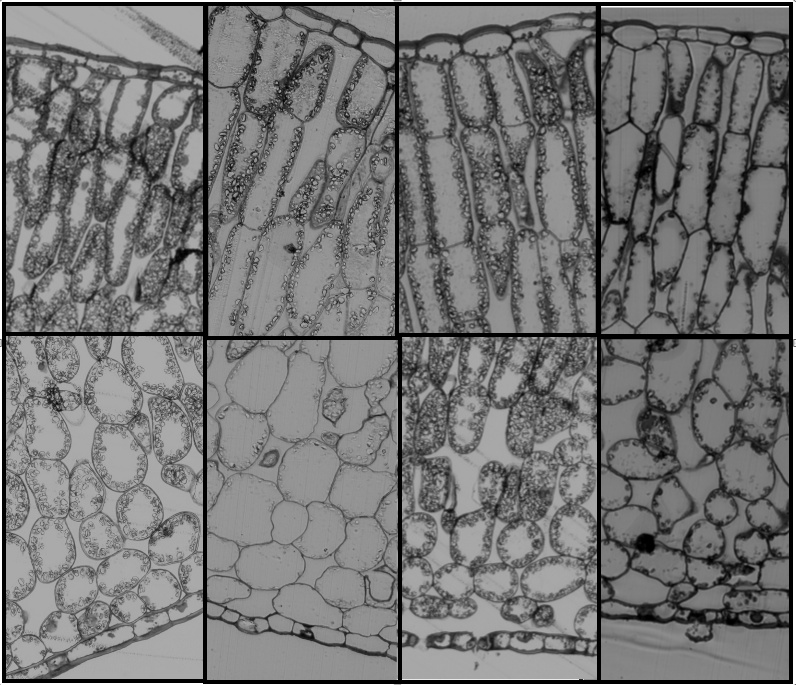


**LE**

**LE**

**LE**

**UE**

**UE**

**UE**

**UE**

**LE**

**h**

**g**

**f**

**e**

**d**

**c**

**b**

**a**

**Supplementary Figure S1:** Micrographs of cross sections from two old leaves of Express genotype in both conditions. a, e : control condition rank 0 ; b, f: Control condition rank -2; c, g: N deficiency condition rank 0; d, h: N deficiency condition rank -1. a, b, c, and d represent the palisade parenchyma while e, f, g, and h the spongy parenchyma. UE: upper epidermis and LE: lower epidermis
