# Supplementary material for: Nitrogen deficiency impacts on leaf cell and tissue structure with consequences for senescence associated processes in Brassica napus
Source: Bot Stud. 2016 May 24;57:11. doi: 10.1186/s40529-016-0125-y (PMC5430559; doi:10.1186/s40529-016-0125-y)
Supplement: Supplementary file 2 — Additional file 2: Figure S2. Developmental stage of Aviso (black) and Express (grey) plants in control (solid line) and N-deficiency (dotted line) conditions. Experiments were conduct on 1/31. [file 40529_2016_125_MOESM2_ESM.docx]

**Supplementary Figure S2:** Developmental stage of Aviso (black) and Express (grey) plants in control (solid line) and N-deficiency (dotted line) conditions. Experiments were conduct on 1/31.
